# High neopterin and IP-10 levels in cerebrospinal fluid are associated with neurotoxic tryptophan metabolites in acute central nervous system infections

**DOI:** 10.1186/s12974-018-1366-3

**Published:** 2018-11-23

**Authors:** Else Quist-Paulsen, Pål Aukrust, Anne-Marte Bakken Kran, Oona Dunlop, Vidar Ormaasen, Birgitte Stiksrud, Øivind Midttun, Thor Ueland, Per Magne Ueland, Tom Eirik Mollnes, Anne Ma Dyrhol-Riise

**Affiliations:** 1 Department of Infectious Diseases, Oslo University Hospital, Ullevaal Hospital, P. O. Box 4956 Nydalen, N-0450 Oslo, Norway; 2 Institute of Clinical Medicine, University of Oslo, Oslo, Norway; 3 Research Institute of Internal Medicine, Oslo University Hospital Rikshospitalet, Oslo, Norway; 4 Section of Clinical Immunology and Infectious Diseases, Oslo University Hospital Rikshospitalet, Oslo, Norway; 5 Faculty of Medicine, University of Oslo, Oslo, Norway; 6 K.G. Jebsen Inflammatory Research Center, University of Oslo, Oslo, Norway; 7 K.G. Jebsen Thrombosis Research and Expertise Center, Tromsø, Norway; 8 Bevital A/S, Bergen, Norway; 9 Department of Microbiology, Oslo University Hospital, Ullevaal, Oslo, Norway; 10 Department of Acute Medicine, Oslo University Hospital, Ullevaal, Oslo, Norway; 11 Department of Immunology, Oslo University Hospital, Oslo, Norway; 12 Department of Clinical Science, University of Bergen, Bergen, Norway; 13 Research Laboratory, Nordland Hospital, Bodø, Norway; 14 Faculty of Health Sciences, University of Tromsø, Tromsø, Norway; 15 Centre of Molecular Inflammation Research, Norwegian University of Science and Technology, Trondheim, Norway

**Keywords:** Encephalitis, Aseptic meningitis, Bacterial meningitis, Cytokines, Chemokines, Kynurenine tryptophan pathway, Indoleamine 2,3-dioxygenase, Neopterin

## Abstract

**Background:**

The host response to intruders in the central nervous system (CNS) may be beneficial but could also be harmful and responsible for neurologic symptoms and sequelae in CNS infections. This immune response induces the activation of the kynurenine pathway (KP) with the production of neuroactive metabolites. Herein, we explored cytokine and KP responses in cerebrospinal fluid (CSF) and serum in patients with encephalitis, aseptic, and bacterial meningitis.

**Methods:**

Cytokines were measured in CSF and serum by multiplex assay in adult patients with encephalitis of infectious, autoimmune or unknown etiology (*n* = 10), aseptic meningitis (ASM, *n* = 25), acute bacterial meningitis (ABM, *n* = 6), and disease control patients with similar symptoms but without pleocytosis in CSF (*n* = 42). Liquid chromatography-tandem mass spectrometry (LC-MS/ MS) was used to measure KP metabolites in CSF and serum.

**Results:**

A characteristic pattern of increasing cytokine levels and KP metabolites was found in CSF from encephalitis to ASM, with the highest levels in ABM. In ASM and ABM, most inflammatory mediators, including IL-6, IL-8, and IFN-inducible protein-10 (IP-10), showed markedly elevated levels in CSF compared with serum, indicating production within the CNS. In contrast to most mediators, the highest level of IP-10 was found in the ASM group, suggesting a potential role for IP-10 in aseptic/viral meningitis. Neopterin and IP-10 were associated with marked changes in KP metabolites in CSF with increasing kynurenine/tryptophan ratio reflecting indoleamine 2,3-dioxygenase activity. Neopterin, a marker of IFN-γ activity, was associated with an unfavorable balance between neuroprotective and neurotoxic tryptophan metabolites.

**Conclusion:**

We show that parenchymal and meningeal inflammations in CNS share a characteristic cytokine profile with a general immune response in the CSF with limited influence from the systemic circulation. IFN-γ activity, assessed by neopterin and IP-10 levels, may play a role in the activation of the KP pathway in these patients, potentially mediating neurotoxic effects.

**Electronic supplementary material:**

The online version of this article (10.1186/s12974-018-1366-3) contains supplementary material, which is available to authorized users.

## Background

The host inflammatory response to intruders to the central nerve system (CNS) plays an important role for neuronal injury in encephalitis and meningitis. The cytokine profiles of aseptic meningitis (ASM) and acute bacterial meningitis (ABM) have been investigated in several studies, in general showing increased levels of inflammatory mediators [1–8]. However, for encephalitis, inflammatory responses have mainly been evaluated for patients with herpes simplex virus (HSV) infection [9–13]. Thus, comparison of cytokine levels in encephalitis, ASM and ABM and control patients are scarce. Moreover, most studies have focused on a limited number of inflammatory markers, and few studies have examined parallel samples of serum and cerebrospinal fluid (CSF) from the same patients.

**Fig. 1:**
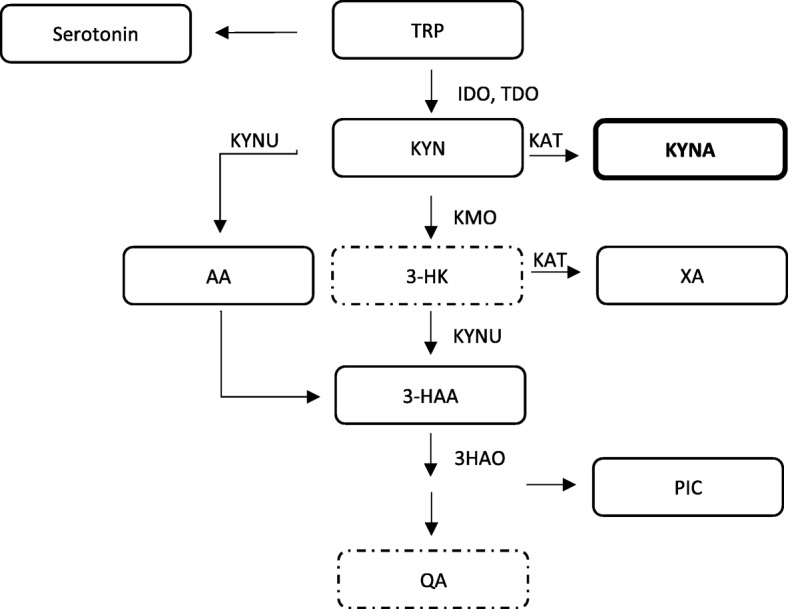
Schematic presentation of the KP pathway. IDO is the main enzyme responsible for the TRP catabolism in CNS. KYN is further degraded into the neuroprotective NMDAr antagonist KYNA by KAT, or by KMO and KYNU into the neurotoxic metabolites of 3-HK and QA. QA is an agonist of the NMDA receptor. Abbreviations: AA, anthranilic acid; 3-HAA, 3-hydroxyanthranilic acid; HAO, 3-hydroxyanthranilic acid oxidase; 3-HK, 3-hydroxykynurenine; IDO, indoleamine-2,3-dioxygenase; KAT, kynurenine aminotransferase; KMO, kynurenine 3-monooxygenase; KYN, kynurenine; KYNA, kynurenic acid; KYNU, kynureninase; QA, quinolinic acid; PIC, picolinic acid; TRP, tryptophan; XA, xanthurenic acid. Bold box indicates neuroprotective metabolite, dashed boxes indicate neurotoxic metabolites in the KP pathway

It is known that the inflammation activates the kynurenine pathway (KP) resulting in the depletion of tryptophan (TRP) and formation of metabolites with potential neurotoxic (e.g., quinolinic acid [QA], 3-hydroxykynurenine [3-HK]) and neuroprotective (e.g., kynurenic acid [KYNA]) effects (Fig. 1) [14, 15]. The activation of the KP seems to also have immune modulating effects, resulting in inhibition of T_H_1 cells, activation of regulatory T cells (T_regs_) and inhibition of natural killer (NK) cells [16–19]. In the CNS, the rate-limiting enzyme for TRP catabolism is indoleamine 2,3-dioxygenase (IDO) which is upregulated by inflammatory cytokines, mainly by interferon gamma (IFN-γ) [20], linking T cell activation to the regulation of the KP.

Altered cytokine levels and associated imbalance of neurotoxic and neuroprotective metabolites in the KP have been suggested to contribute to the pathogenesis of several chronic conditions in the CNS, such as schizophrenia [21, 22], bipolar disorders [23, 24], Parkinson’s disease [25], Alzheimer’s [26] and Huntington’s disease [27], and AIDS-related dementia [28, 29], as well as in traumatic brain injury [30]. However, there is limited data on the role of KP in acute infections like encephalitis and meningitis, although increased levels of KYNA have been shown in patients with HSV encephalitis, Lyme borreliosis, tick-borne encephalitis, and bacterial meningitis [4, 31–33]. Moreover, altered tryptophan metabolism has been linked to disease severity in tuberculous meningitis [34].

The aim of the present study was to elucidate the inflammatory network and KP metabolites in ASM and ABM, characterized by meningeal inflammation, and in encephalitis, characterized by brain parenchymal inflammation. Parallel samples of serum and CSF were examined in patient groups and control patients, i.e., patients with similar symptoms but without CSF pleocytosis or other signs of CNS infection.

## Methods

### Study participants and study design

This cross-sectional study was performed at the Oslo University Hospital (OUS), Ullevaal, a regional hospital for 2.7 million people. Patients were eligible for inclusion if they had (1) onset of symptoms of CNS infection within less than 7 days and (2) clinical indication for a diagnostic lumbar puncture (LP). Patients were included between January 2014 and December 2015. CSF leukocyte counts ≥ 5 × 10^6^ /L was found in 68 patients. Of these, 23 did not fulfill the case definition of encephalitis of viral, autoimmune or unknown cause, aseptic meningitis (ASM), or bacterial meningitis (ABM) (Additional file 1: Table S1). In four patients, the time from CSF sampling until centrifugation was > 10 h, rendering a total of 41 patients with CNS infection (Additional file 2: Figure S1). The control group consisted of age- and gender-matched patients with similar symptoms, but without signs of CNS inflammation, i.e., no pleocytosis and no microbiological agent detected in their CSF (*n* = 42). In the control group, patients with delirium, chronic or acute psychiatric disease, Parkinson’s disease, Huntington’s disease, CNS malignancy, dementia, epilepsy or seizures, cerebral vascular disease, transient global amnesia, and septicemia were not included. For detailed case definitions, see Additional file 1: Table S1. Flowchart of the study population and the overview of analyses performed for the various groups are shown in Additional file 2: Figure S1.

All patients, or their next of kin when the patient was not able to consent, gave written informed consent to participate in the study. The study was approved by The Regional Committees for Medical and Health Research Ethics (REC South East, reference number 2011/2578) and the ethical council of the hospital.

### Microbiological diagnostics

For all included patients, CSF leukocyte counts (CSF WBC), CSF protein, and CSF glucose were measured. Bacterial culture of CSF and analyses for identification of causative agent were performed in all individuals by a panel of specific PCR for common neurotropic virus and bacteria.

### Sampling of CSF and serum

CSF samples were collected in endotoxin-free polypropylene tubes and stored at 4 °C until centrifugation at 2000×*g* for 10 min. Serum samples were collected in endotoxin-free tubes without any additives, allowed to clot at room temperature, and centrifuged at 3000×*g* for 10 min. Supernatants from CSF and serum were centrifuged within 10 h after collection and immediately frozen in triplicates of approximately 700 μL each at − 80 °C. All analyses were performed on previously unthawed samples. For six patients, no serum was available.

### Multiplex analyses of soluble markers in CSF and serum

A multiplex cytokine assay (Bio-Plex Pro Human Cytokine 27-plex Panel; Bio-Rad laboratories Inc. Hercules, CA) was used to measure the concentrations of 27 different cytokines: tumor necrosis factor (TNF), IFN-γ, interleukin (IL)-1β, IL-1 receptor antagonist (IL-1Ra), IL-2, IL-4, IL-5, IL-6, IL-7, IL-8/CXCL8, IL-9, IL-10, IL-12(p70), IL-13, IL-15, IL-17A, monocyte chemoattractant protein (MCP)-1/CCL2, IFN-inducible protein-10 (IP-10)/CXCL10, eotaxin/CCL11, macrophage inflammatory protein-1α and -1β (MIP-1α/CCL3, MIP-1β/CCL4), regulated on activation, normal T cell expressed and secreted (RANTES/CCL5), granulocyte-colony stimulating factor (G-CSF), granulocyte-macrophage CSF (GM-CSF), basic fibroblast growth factor (FGF), platelet-derived growth factor (PDGF), and vascular endothelial growth factor (VEGF). The assay was performed using the instructions of the manufacturer. CSF samples were tested undiluted. Only cytokines with less than 20% missing values were included in further analyses. Undetectable levels were assigned the lowest detectable level (LDL) measured in the cohort for the respective marker. In the CSF samples, IFN-γ, IL-5, PDGF, Basic FGF, and RANTES, and for serum, IL-2, IL-5, IL-7, IL-15, G-CSF, GM-CSF, and FGF were excluded from further analyses based on the criteria stated above.

### Mass spectrometry analyses of tryptophan metabolites in CSF and serum

Tryptophan and kynurenine metabolites were measured only in the patients with encephalitis, ASM with confirmed viral etiology (viral meningitis, VM), ABM, and controls (Additional file 2: Figure S1). Concentrations of TRP, kynurenine [KYN], anthranilic acid [AA], KYNA, 3-HK, 3-hydroxyanthranilic acid [3-HAA], xanthurenic acid [XA], QA, picolinic acid [PIC], and neopterin were analyzed in CSF and serum by liquid chromatography-tandem mass spectrometry (LC-MS/MS) by Bevital AS [35, 36]. For TRP, nine patients had levels in CSF below the lower limit of detection (LOD). As this may represent a finding rather than a limitation by the analysis, these were set equal to the LOD (0.4 μM) in the statistical analyses and in the calculation of the KYN/TRP ratio (KYN (nmol)/TRP (μmol)) as an index of IDO activity. XA was not detected in CSF for 46 of the 50 patients and was not included in the analysis.

### Statistical methods

Continuous data are presented as median (IQR, interquartile range). Due to lack of normal distribution, analysis of variance (ANOVA) with the Kruskal-Wallis test for multiple groups was used. If the Kruskal-Wallis test revealed significant differences, the Mann-Whitney *U* test was used to compare pairs of groups. To limit type II statistical errors, no correction for multiple comparisons was made in this explorative study. *P* values < 0.05 were considered statistically significant. Categorical variables are expressed as counts (percentages) and analyzed by Pearson’s chi-square test. Correlations were analyzed using Spearman’s rank correlation coefficient. All data analyses were performed in SPSS version 24 (IBM Corp. Armonk, NY, USA) and graphs generated by GraphPad Prism 7 (GraphPad, San Diego, USA).

## Results

### Study participant characteristics

Ten patients had encephalitis of viral, autoimmune, or unknown etiology according to the case definition (Additional file 1: Table S1), 25 patients were diagnosed with ASM, six patients with ABM, and 42 were control patients. Characteristics of the study group are presented in Table 1. There were no significant differences in gender or age between the patient groups. In the CNS infection group, four patients reported a history of depression, but only two of these received antidepressant drugs. The etiology of encephalitis was known for four patients (40%), three viral (adenovirus, HSV1, varicella-zoster virus [VZV]) and one N-methyl-D-aspartate receptor [NMDAr] encephalitis. Of the 25 patients with ASM, eight were diagnosed with enterovirus in CSF, six patients suffered from HSV2 meningitis, one patient seroconverted and had positive IgM in CSF for Toscana virus, and for one patient, intrathecal antibody production of IgG against Borrelia burgdorferi was detected. For patients with ABM, *Streptococcus pneumoniae* (*n* = 2), *Staphylococcus aureus* (*n* = 2), *Neisseria meningitidis* (*n* = 1), and *Haemophilus influenzae* (*n* = 1) were detected in CSF by growth and/or PCR, and for all these patients, the causative bacteria was also detected in blood culture. Patients with encephalitis presented with less headache and more focal neurology compared to the other groups, and impairment of consciousness was observed in significantly fewer patients with ASM and in the control group. Importantly, the majority of the control group had fever, headache, and neck stiffness, similar to most of the patients with CNS infection.

**Table 1:** Patient characteristics and clinical presentation

Parameter	Encephalitis (*n* = 10)	ASM (*n* = 25)	ABM (*n* = 6)	Controls (*n* = 42)	*p* value^a^
Gender, males (%)	4 (40)	10 (40)	4 (67)	13 (31)	0.385
Age, years	43.5 (30, 72)	35 (28, 48)	52 (41, 68)	31 (22, 41)	0.054
Hospital stay, days	19 (11, 42)^b^	3 (1.5, 5.5)^c^	19 (14, 33)^b, d^	2.0 (1.0, 4.0)	**< 0.001**
Comorbidity (%)					
Immunodeficiency^e^	2 (30)^b^	1 (4)^c^	1 (17)^b^	–	**0.029**
Psychiatric disorder	2 (20)^b^	2 (8)	–	–	**0.046**
Etiology known (%)^f^	4/10 (40)	16/25 (64)	6/6 (100)	–	**< 0.001**
Headache (%)	7/10 (70)^b^	24/25 (96)^c^	3/4 (75)	39/41 (95)	**0.037**
Neck stiffness (%)^g^	2/10 (20)	12/25 (48)	3/5 (60)	12/42 (29)	0.175
Objective fever (%)^h^	7/10 (70)	17/25 (68)	5/6 (83)	20/42 (48)	0.168
Focal neurology (%)	5/9 (56)^b^	2/19 (10)^c^	1/6 (17)	1/24 (4)	**0.003**
GCS ≤ 14 (%)	10/10 (100)^b^	2/25 (8)^c^	5/6 (83)^b,d^	6/42 (14)	**< 0.001**
CSF WBC (× 10^6^/L)	25 (9.5, 92)^b^	179 (26, 271)^b, c^	212 (91, 1434)^b, c^	1.0 (1.0, 2.0)	**< 0.001**
CSF protein (g/L)	0.57 (0.4, 0.9)^b^	0.59 (0.4, 0.8)^b^	2.2 (0.8, 5.9)^b, c, d^	0.26 (0.2, 0.3)	**< 0.001**
CSF glucose (mmol/L)	3.6 (3.3, 4.4)	3.5 (3.0, 3.7)	3.6 (0.4, 6.3)	3.5 (3.2, 3.7)	0.341
Glucose ratio	0.6 (0.5, 0.7)	0.6 (0.5, 0.6)^b^	0.4 (0.1, 0.6)^b^	0.6 (0.6, 0.7)	**0.009**
Albumin ratio	8.4 (6.5,14)^b^	9.4 (6.1, 13)^b^	79 (36, 137)^b, c, d^	4.0 (2.6, 5.0)	**< 0.001**
Blood WBC (× 10^9^/L)	8.6 (7.4, 11)	8.2 (6.1, 11)	14 (8.5, 18)	10 (6.7, 12)	0.173
CRP, serum (mg/L)	6 (0.9, 86)	3.5 (1.3, 12)^b^	169 (96, 446)^b, c, d^	16 (2.1, 77)	**0.001**

*CSF WBC* white blood cell count in CSF, *glucose ratio* CSF glucose/serum glucose, *albumin ratio* CSF albumin/serum albumin. Significant p-values are marked in bold.

Data shown are median (IQR) or numbers/*n* (%)

^a^*p* values for one way analysis of variance (Kruskal-Wallis)

^b^*p* < 0.05 for analysis with Mann-Whitney *U* test (MWU) in comparison with the control group

^c^*p* < 0.05 for analysis with MWU in comparison with encephalitis

^d^*p* < 0.05 for analysis with MWU in comparison with ASM

^e^Under treatment for or treated for cancer within the last year (including hematological malignancies), HIV infection or diabetes mellitus type 2 (DM2), or using immunosuppressive or immune modulating drugs

^f^Causing agents for encephalitis were 3 viral (adenovirus, HSV1, VZV) and 1 NMDAr encephalitis. ABM; *Streptococcus pneumonia* (*n* = 2), *Staphylococcus aureus* (*n* = 2), *Neisseria meningitides* (*n* = 1), and *Haemophilus influenzae* (*n* = 1). ASM; HSV2 (*n* = 6), enterovirus (*n* = 8), Toscana virus (*n* = 1), and 1 patient had intrathecal antibody production of IgG against *Borrelia burgdorferi*

^g^Neck stiffness was assessed by a physician before LP

^h^Fever was defined as either ≥ 38 °C upon admission or within 24 h after admission, or measured to ≥ 38 °C by the patient prior to admission

### Cytokine profiles in CSF and serum

**Fig. 2:**
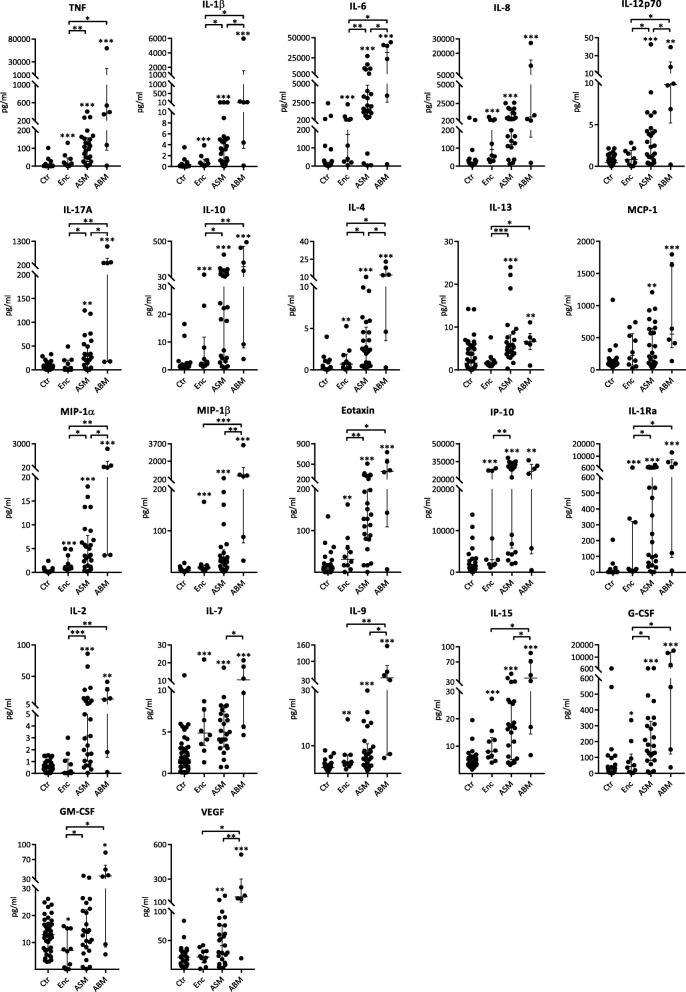
Cytokines in CSF in patients with encephalitis (Enc, *n* = 10), aseptic meningitis (ASM, *n* = 25), bacterial meningitis (ABM, *n* = 6) in comparison with controls (Ctr, *n* = 42). Data shown are medians with IQR and all were significant by the Kruskal-Wallis test. Comparisons of two groups were analyzed by using Mann-Whitney *U* test. Asterisks above patient groups indicate significant difference vs controls, asterisks above horizontal lines indicate significant differences between individual groups (Mann-Whitney *U* test): *p < 0.05, **p < 0.01, and ***p < 0.001. Values below the detection limit were set to the lowest detectable level for that analyte

Cytokines analyzed in parallel in CSF and serum showed distinct patterns for the different patient groups. Overall, the highest levels of CSF cytokines were found in patients with ABM, including the prototypical inflammatory cytokines TNF, IL-1β and IL-6, inflammatory chemokines (e.g., IL-8, MCP-1, MIP-1α and MIP-1β), cytokines with potent effect on T cell function (e.g., IP-10, IL-7, IL-9 and IL-15), and growth factors (e.g., VEGF and G-CSF) (Fig. 2, Additional file 3: Table S2). The typical cytokine pattern in CSF was an increase from disease controls without CNS infection to patients with encephalitis and ASM with the highest levels in those with ABM. In contrast to this pattern, the CXC chemokine IP-10 showed the highest median level in the ASM group (Fig. 2). Although lower than in patients with ABM and ASM, patients with encephalitis had higher levels for most cytokines in CSF compared to the control group, with no significant difference in the levels between encephalitis cases with or without known etiology (data not shown).

**Fig. 3:**
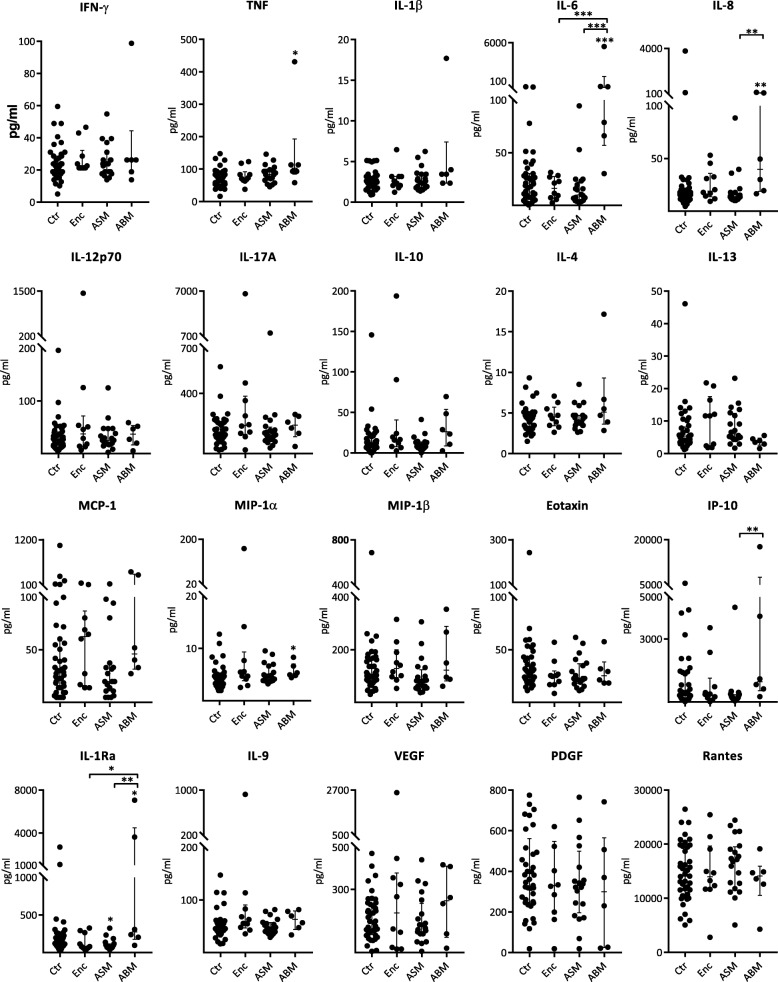
Cytokines in serum in patients with encephalitis (Enc, *n* = 10), aseptic meningitis (ASM, *n* = 20), bacterial meningitis (ABM, *n* = 6) in comparison with controls (Ctr, *n* = 41). Data shown are medians with IQR. Asterisks above patient groups indicate significant difference vs controls, asterisks above horizontal lines indicate significant differences between individual groups (Mann-Whitney *U* test): *p < 0.05, **p < 0.01, and ***p < 0.001. Values below the detection limit were set to the lowest detectable level for that analyte

The encephalitis group and the control group generally showed lower cytokine levels in CSF than in serum, with exceptions for IL-6 and IL-8 in the encephalitis group and MCP-1 and IP-10 for both groups (Figs. 2 and 3, Additional file 3: Table S2). In contrast, the groups with meningeal involvement and in particular the ABM group displayed markedly higher CSF levels than serum levels for most cytokines. For some of the cytokines, the CSF levels were more than tenfold higher than in serum (e.g., IL-6, IL-8, IP-10). In general, serum levels did not display the striking and significant differences between the patient groups and controls as seen in CSF (Fig. 3). In fact, although all patients in the ABM group had a positive blood culture, only TNF, IL-6, IL-8, IL-1Ra, and MIP-1α demonstrated higher serum levels than in the control group.

When we analyzed the CNS infections all together (encephalitis, ASM, and ABM), a significant correlation between serum and CSF levels were found only for TNF (Rho 0.4, *p* = 0.03), IL-1Ra (Rho 0.4, *p* = 0.03), IL-6 (Rho 0.5, *p* = 0.004), and MCP-1 (Rho 0.3, *p* = 0.04) suggesting intrathecal production of most of the examined mediators (Additional file 4: Table S3). Finally, except for MCP-1, all cytokines in CSF correlated positively with the CSF leukocyte counts, and all, except for MCP-1 and IP-10, correlated with CSF/serum albumin ratio. Collectively, these data underscore the limited information obtained from a serum sample in contrast to that obtained from CSF when examining the inflammatory network in infectious CNS diseases.

### Profile of tryptophan metabolites and IDO activity in CNS infections

**Fig. 4:**
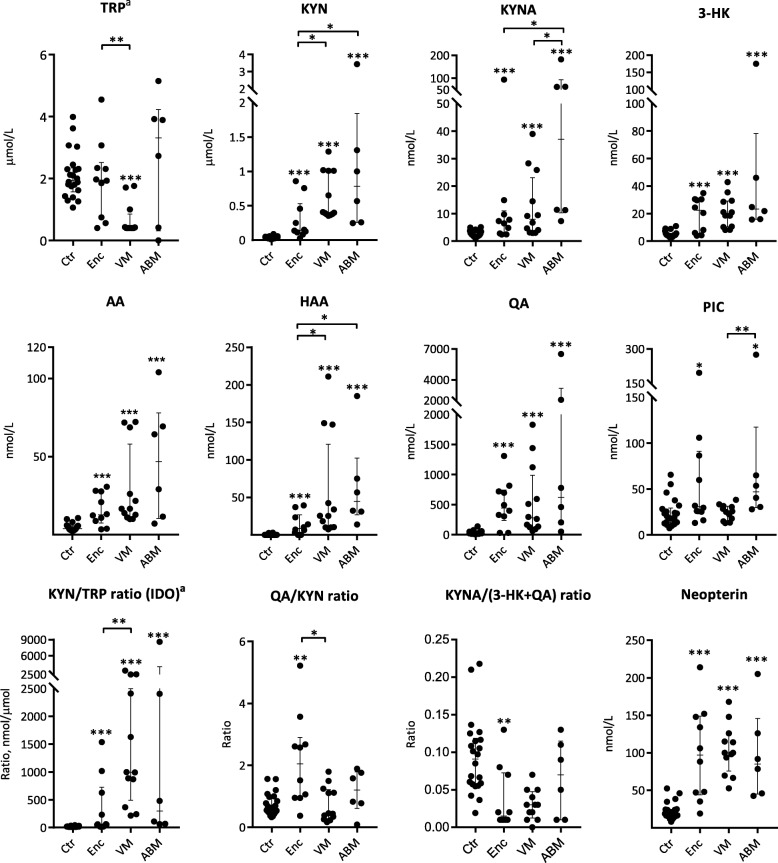
Neopterin, kynurenine metabolites, and ratios in patients with encephalitis (Enc, *n* = 10), viral meningitis (VM, *n* = 12), and bacterial meningitis (ABM, *n* = 6) in comparison with controls (Ctr, *n* = 22). Data shown are median with IQR, and all were significant in the analysis of variance with the Kruskal-Wallis test. Comparisons of two groups were analyzed by Mann-Whitney *U* test. Asterisks above patient groups indicate significant difference vs controls, asterisks above horizontal lines indicate significant differences between individual groups (Mann-Whitney *U* test): *p < 0.05, **p < 0.01, and ***p < 0.001. a: TRP levels below the lower level of detection (LOD) for 9 patients with CNS infection were adjusted to this value (0.4 μM) for calculation of the KYN/TRP ratio as the expression of IDO activity (KYN (nmol)/TRP(μmol))

In order to relate the cytokine profile to tryptophan metabolism (Fig. 1), metabolites of the KP in the CSF were examined in patients with encephalitis (*n* = 10), those in the ASM group with verified viral cause (VM, *n* = 12), ABM (*n* = 6), and controls (*n* = 22). The median levels of most KP metabolites were higher in patients with encephalitis, VM, and ABM compared with the controls, with the highest median levels observed in the ABM group (Fig. 4, Additional file 5: Table S4). The only exception was TRP, which was lowest in the VM group (Fig. 4).

The KYN/TRP ratio was higher in all CNS infections compared to the controls (*p* = 0.009), indicating an increased conversion of TRP (Fig. 4). Moreover, to evaluate the relationship between putatively neuroprotective (KYNA) and neurotoxic (3-HK and QA) KP metabolites, we calculated the KYNA/(3-HK + QA) ratio showing decreased levels for patients with encephalitis indicating a net neurotoxic effect of TRP metabolites in these patients (Fig. 4).

Most KP metabolites were present at higher concentrations in serum (Additional file 5: Table S4, Additional file 6: Figure S2) than in CSF (Fig. 2), but with less significant differences between the groups. For the total group with CNS infection (encephalitis, VM and ABM), CSF levels correlated with serum levels for 3-HK (Rho 0.6, *p* < 0.001), QA (0.4, *p* = 0.04), and PIC (0.7, *p* < 0.001), for all other KP metabolites, there was no significant correlation between CSF and serum levels. Furthermore, all CSF/serum ratios for KP metabolites, except for TRP, KYNA, and PIC ratio, were positively correlated with CSF WBC count, while only the ratio of PIC was correlated with CSF albumin/serum ratio (Additional file 7: Table S5).

### Markers of IFN-γ activation correlate with activation of the KP

**Fig. 5:**
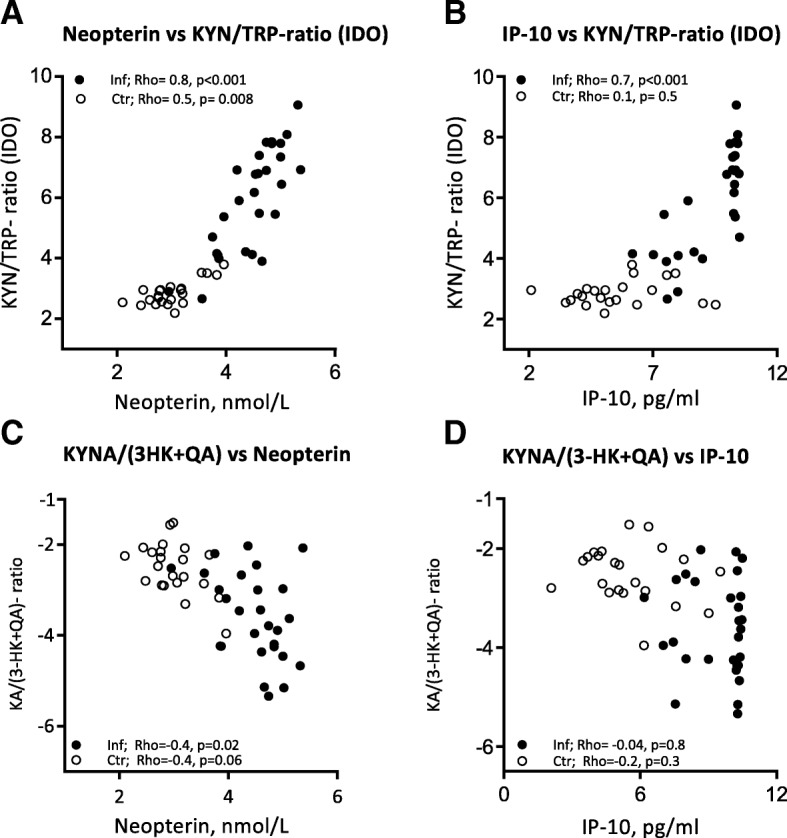
Correlations of neopterin and IP-10, as markers of IFN-ƴ activity, with activation of the KP. **a** Neopterin vs KYN/ TRP ratio (IDO) (*n* = 50; Rho 0.9, *p* < 0.001). **b** IP-10 vs KYN/TRP ratio (IDO) (*n* = 50; Rho 0.8, *p* < 0.001). **c** Neopterin vs KYNA/(3-HK + QA) ratio (*n* = 50; Rho − 0.7, *p* < 0.001). **d** IP-10 vs KYNA/(3-HK + QA) ratio (*n* = 50, Rho − 0.5, *p* < 0.001). Data shown are obtained by Spearman’s rank correlation

IFN-γ is known to have a major influence on the KP [37, 38]. In the present study, IFN-γ levels in CSF were below detection level in 58% of the cases. However, neopterin, being a reliable and stable marker of IFN-γ activity [39], was markedly elevated in CSF in all groups of patients with neuroinflammation compared with controls (Fig. 4). Notably, when all CNS infections were analyzed together, CSF level of neopterin was strongly positively correlated with IDO (measured as the KYN/TRP ratio) and inversely correlated with the KYNA/(3-HK + QA) ratio (Fig. 5). This suggests an association between high IFN-γ activity and net neurotoxic effects of KP metabolites. A positive correlation was also seen between IDO and IP-10, another IFN-ƴ related cytokine, but no significant correlation was found between IP-10 and KYNA/(3-HK + QA) ratio (Fig. 5).

### Association of cytokines and metabolites in the KP and clinical variables

Abnormal regulation of the KP metabolites has been related to depression [40, 41]. However, excluding the two patients in the encephalitis group who reported existing or recent depression from the correlation analyses did not influence the findings (data not shown). Although the Glasgow Coma Scale (GCS) is a crude tool, it reflects the severity of critically ill patients. Among patients with aseptic meningitis, all but two had a GCS at 15; thus, only patients with encephalitis and bacterial meningitis were included in correlation analyses with GCS. We did not find any significant correlations between cytokine levels or KP metabolites in CSF and GCS in these patients (data not shown). However, the number of patients was too low to make any firm conclusion.

## Discussion

In the present study, we demonstrate a gradual increase in levels of a wide range of cytokines, including chemokines, interleukins, interferons, and growth factors from encephalitis to ASM and finally to ABM as compared with controls, reflecting the level of CSF inflammation seen in patients with these CNS infections. Moreover, in ASM and ABM, the levels were much higher in CSF than in serum for most of the mediators, even though the patients with ABM had positive blood cultures for the actual pathogen. Notably, in contrast to most of the mediators, IP-10 levels in CSF had the highest median value in the ASM group, indicating a potential role for this chemokine in aseptic meningitis. Finally, these changes in inflammatory mediators were associated with marked changes in KP metabolites in CSF. In particular, all groups of CNS infections had increased IDO activity assessed by KYN/TRP ratio compared with controls, indicating an increased catabolism of TRP. Interestingly, patients with encephalitis and viral meningitis had an unfavorable balance between neuroprotective and neurotoxic TRP metabolites. These changes in KP metabolites were associated with CSF levels of neopterin, and for the KYN/TRP ratio also with IP-10, suggesting a link between IFN-γ and altered KP metabolites in these patients.

The influx of inflammatory cells and the resulting dysregulation of cytokine networks may be detrimental during CNS infection and is thought to contribute to neurological complications [12, 42–44]. CNS inflammation is a complex process, depending on anatomical site (parenchyma vs meninges), cell type being involved (e.g., infiltrating leukocytes in acute infection vs brain-derived cells), and causing agent (e.g., viral vs bacterial). In this study, we describe findings from patients with acute inflammation in the CNS, mainly of infectious cause. The levels of cytokines in CSF were generally higher for all infectious groups compared to control, and strongly correlated with each other, demonstrating a general inflammatory response in patients with acute CNS infections. The pattern of increasing levels of inflammatory markers from encephalitis to aseptic meningitis and finally to bacterial meningitis indicates increased inflammation in all subgroup of patients, but with a more modest CSF inflammation in patients with encephalitis. Not surprisingly, the highest levels of most of the mediators were found in patients with bacterial meningitis and there was a CSF/serum ratio > 1 of most mediators in patients with ABM, despite bacteremia. Furthermore, most cytokines were correlated with CSF WBC count, but not with corresponding serum levels, which implies the inflammatory response in the CNS to be independent of a peripheral immune response with no or minimal influx or efflux between the two compartments. These results indicate a predominately intrathecal production of these mediators in CNS infections. IL-6, IL-8, and TNF in CSF have in several studies been found to be elevated in meningitis and have even been proposed as biomarkers in CSF for bacterial meningitis [6, 45]. We found a general increase for most of the inflammatory mediators in both ASM and ABM, and our study does not support the use of one particular cytokine as a diagnostic marker to distinguish ABM from other CNS conditions, including ASM.

In contrast to most of the inflammatory markers, IP-10 levels were comparable in patients with ASM and ABM. IP-10 is secreted by several cell types in response to IFN-γ, and experimental studies have shown that IP-10 is highly induced in the CNS (e.g., West Nile infections [46], HIV encephalitis [47], HSV encephalitis [48], enteroviral encephalitis [3, 49], Japanese encephalitis [50], and tick-borne encephalitis [TBE] [51]). Increased levels have also been reported in patients suffering from TBE [52], neuroborreliosis [53], enterovirus infection [3], HSV meningitis, and HSV encephalitis [9]. IP-10 is a chemoattractant for monocytes and macrophages and promotes T cell recruitment, especially of CD8^+^ T cells [46, 54, 55]. Although IP-10 has been linked to viral clearance in the CNS [48, 55], IP-10 has also been described to promote apoptosis of neurons and excessive production has been associated with more severe outcome [47, 50, 51]. In the present study, we found an association between IP-10 and the KYN/TRP ratio, indicating increased IDO activity and catabolism of TRP with potential harmful effects on CNS. Thus, our present data may further support a potential role for this chemokine in CNS infections, particularly in ASM. This should be further investigated.

Neurologic dysfunction is common in patients with encephalitis and bacterial meningitis, and dysregulation of the KP has been shown in syndromes and disorders with certain overlap in symptoms [21, 30, 32, 56, 57]. Herein, we compared these metabolites in patients with a stringent definition of etiology; encephalitis, confirmed viral meningitis, and bacterial meningitis. Recent literature has shown that in HSV encephalitis, a low level of the neuroprotective metabolite KYNA was associated with more severe outcome [31]. In TB meningitis, low TRP was associated with a better survival rate [34]. In our study, the ratio of the neuroprotective metabolite KYNA to the sum of neurotoxic metabolites 3-HK and QA was lower for patients with encephalitis compared to the other groups, which could indicate a stronger activation of the KMO branch in encephalitis. In fact, studies are ongoing regarding the inhibition of several steps in the KP, including studies on centrally available KMO inhibitors [58].

In the present study, we observed very low levels of TRP for several patients with CNS infection, especially in patients with ASM. We hypothesize that this results from increased IDO activity, as these patients had a significantly higher level of KYN compared to patients with detectable TRP levels. The strong correlation of KYN/TRP ratio with neopterin and IP-10 indicates that this IDO activity is driven by IFN-γ. IDO has been found to exhibit an immunosuppressive effect by upregulation of T_regs_ and downregulation of Th17 cells, which could be relevant in CNS infections, especially in ASM where T cells are of particular importance. In contrast to the association with IDO activity, neopterin, but not IP-10, was associated with a “neurotoxic” KYNA/(3-HK + QA) ratio in patients with CNS infection. The lack of correlation of IP-10 to KYNA/(3-HK + QA) could be related to low statistical power. However, whereas IP-10 is induced by IFN- γ in several cell types including monocytes, stromal cells, and endothelial cells [59], neopterin is almost selectively produced in monocytes/macrophages, and through its relation to tetrahydrobiopterin, neopterin may be more closely related to tryptophan metabolism than IP-10 [60, 61].

Finally, when looking at the CSF/serum ratios of KP metabolites, we found very narrow ranges in the control group, suggesting strict control of KP in healthy subjects.

Studies of the inflammatory profile in human CNS infections including both meningeal and parenchymal infections are relatively scarce [62–65], and comparisons between different studies are hampered by the diversity of causing agents and divergent inclusion criteria. Nevertheless, knowledge of immunological mechanism is pivotal in order to develop better diagnostic and potentially therapeutic tools for these patients. To our knowledge, the measurement of a large panel of metabolites in the KP in both serum and CSF, with parallel analyses of a wide range of cytokines and related mediators, have not been reported for patients with these conditions, especially not for encephalitis. Moreover, the correlation in present study of IP-10 and neopterin with the KP metabolites and the decreased KYNA/(3-HK + QA) ratio in encephalitis are interesting findings that, as far as we are aware of, have not been reported in these clinical relevant CNS infections. However, the present study also has some limitations. The subgroups of patients, and in particular patients with ABM and encephalitis, were small and too small for sub-analyses on causing agents. Moreover, the etiology was unknown for 60% of patients with encephalitis with few patients with confirmed viral cause. Besides, due to lack of reliable measures of severity and outcome, together with the relatively low number of patients with CNS infection, we cannot make any conclusion regarding the use of these cytokines and metabolites as prognostic markers. The control group in our study consisted of patients with similar presentation and no pleocytosis, but many of these patients suffered from systemic infections, as reflected by elevated serum neopterin levels. This may have camouflaged significant findings in serum profiles. On the other hand, these controls may be more clinically relevant than healthy controls without any disease symptoms like fever and headache. Finally, correlations do not necessarily mean any causal relationship and more mechanistic studies as well as larger studies with samples also taken during follow-up are needed to make firmer conclusions.

## Conclusions

In conclusion, we found a marked increase in a wide range of inflammatory mediators in CSF in aseptic and bacterial meningitis with a more modest increase in encephalitis. The markedly higher levels in CNS than in serum for most of the mediators indicate compartmentalization of immune responses in the CSF. Our data may also suggest that increased IFN-γ activity, as assessed by neopterin and IP-10, may contribute to neurotoxicity through enhanced TRP catabolism. In particular, dysregulation of the KP with signs of an increased formation of neurotoxic QA in encephalitis should be explored further in these conditions.

## Additional files

Additional file 1: **Table S1.** Case definitions of encephalitis, aseptic and viral meningitis and bacterial meningitis. (PDF 179 kb)

Additional file 2: **Figure S1.** Flowchart of inclusion of patients and overview of various analyses performed in the study population. (PDF 342 kb)

Additional file 3: **Table S2.** Cytokine levels in CSF and serum. (PDF 215 kb)

Additional file 4: **Table S3.** Correlations of cytokines in CSF with serum levels, CSF WBC, albumin ratio and KYN/TRP ratio. (PDF 197 kb)

Additional file 5: **Table S4.** KP metabolites in CSF and serum. (PDF 198 kb)

Additional file 6: **Figure S2.** Serum levels of KP metabolites. (PDF 81 kb)

Additional file 7: **Table S5.** CSF/serum ratios of KP metabolites and the correlation with CSF WBC and albumin ratio. (PDF 194 kb)

## Abbreviations

3-HAA: 3-Hydroxyanthranilic acid
3-HK: 3-Hydroxykynurenine
AA: Anthranilic acid
ABM: Acute bacterial meningitis
Albumin ratio: CSF albumin/serum albumin
ASM: Aseptic meningitis
Glucose ratio: CSF glucose/serum glucose
HAO: 3-Hydroxyanthranilic acid oxidase
HSV1: Herpes simplex 1 virus
IDO: Indoleamine 2,3-dioxygenase
KAT: Kynurenine aminotransferase
KMO: Kynurenine 3-monooxygenase
KP: Kynurenine pathway of tryptophan metabolism
KYN: Kynurenine
KYNA: Kynurenic acid
KYNU: Kynureninase
LDL: The lowest detectable level
LOD: Lower limit of detection
NMDAr: N-methyl-D-aspartate receptor
QA: Quinolinic acid
TRP: Tryptophan
VM: Viral meningitis
VZV: Varicella-zoster virus
WBC: White blood cell count
XA: Xanthurenic acid
