# Supplementary material for: High neopterin and IP-10 levels in cerebrospinal fluid are associated with neurotoxic tryptophan metabolites in acute central nervous system infections
Source: J Neuroinflammation. 2018 Nov 23;15:327. doi: 10.1186/s12974-018-1366-3 (PMC6260858; doi:10.1186/s12974-018-1366-3)
Supplement: Supplementary file 1 — Table S1. Case definitions of encephalitis, aseptic and viral meningitis and bacterial meningitis. (PDF 179 kb) [file 12974_2018_1366_MOESM1_ESM.pdf]

Table S1 Case definitions

Condition	Case definition
Encephalitis[a]	Encephalopathy (altered mental function or change in personality) for >24 hours with no other cause identified and at least two of the following:<br>1. ≥5 x10^6/L leucocytes (WBC) in cerebrospinal fluid (CSF)<br>2. new onset of seizures,<br>3. new onset of focal neurology,<br>4. documented fever >38°C before or within 24 hours after hospitalisation,<br>5. EEG findings suggestive of encephalitis and/or<br>6. MRI/CT findings suggestive of encephalitis
Aseptic meningitis (ASM)	1. Clinical signs of meningitis (headache, neck stiffness, photophobia and/or fever),<br>2. ≥5 x10^6/L leucocytes in CSF and<br>3. culture negative CSF
Acute bacterial meningitis (ABM)	1. Clinical signs of meningitis (as criteria 1 for ASM),<br>2. ≥5 x10^6/L leucocytes in CSF and<br>3. detected bacteria in CSF, either by culture or PCR
Viral meningitis (VM)	1. As aseptic meningitis, criteria 1 and 2 and<br>2. virus detected by PCR in CSF

[a]based on the Consortium Definition published in 2013 by Venkatesan et al; CID 2013:57
