# Supplementary material for: High neopterin and IP-10 levels in cerebrospinal fluid are associated with neurotoxic tryptophan metabolites in acute central nervous system infections
Source: J Neuroinflammation. 2018 Nov 23;15:327. doi: 10.1186/s12974-018-1366-3 (PMC6260858; doi:10.1186/s12974-018-1366-3)
Supplement: Supplementary file 2 — Figure S1. Flowchart of inclusion of patients and overview of various analyses performed in the study population. (PDF 342 kb) [file 12974_2018_1366_MOESM2_ESM.pdf]

Fig. S1 Overview of inclusion of patients

A.

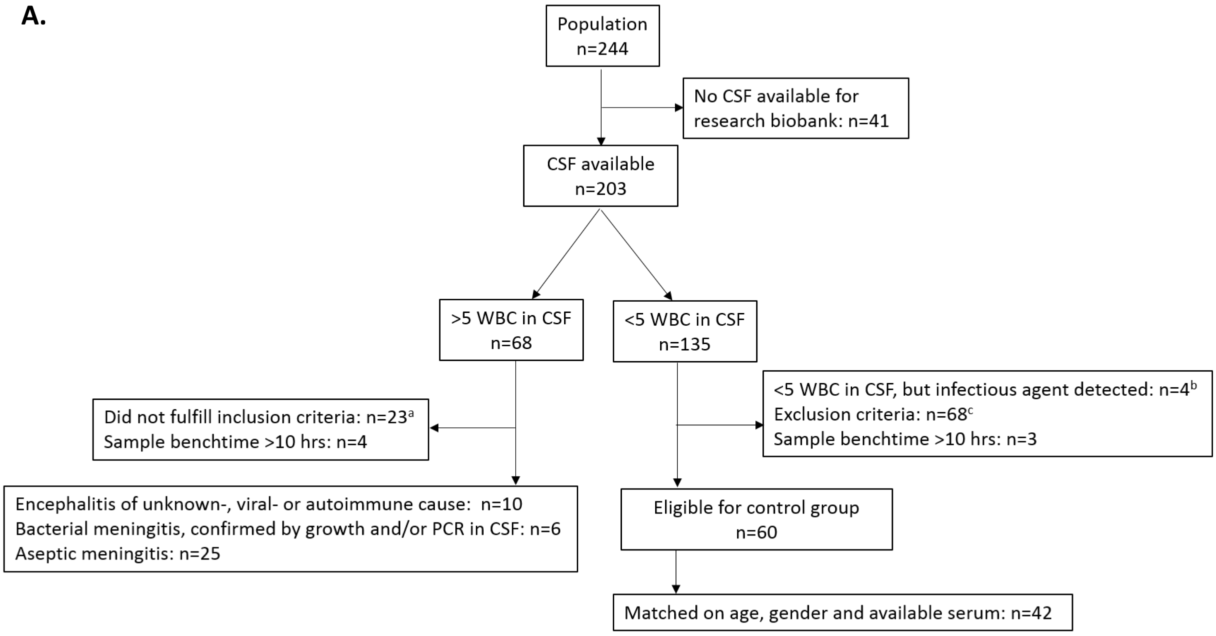

B.

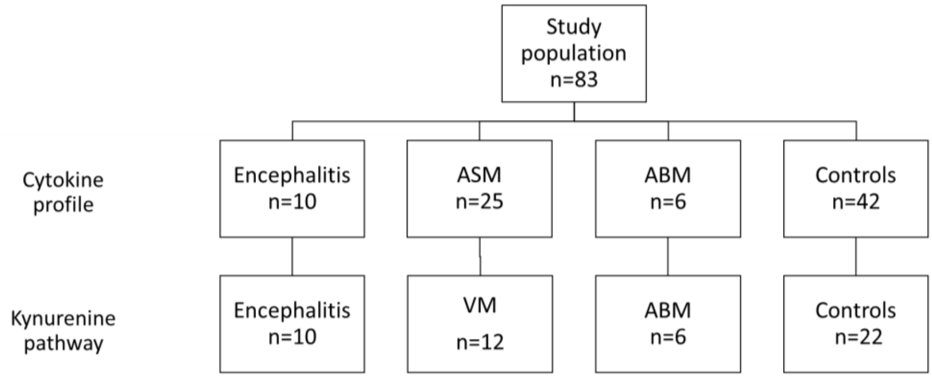

A. Flow chart of inclusion of patients to the present study

[a]patients with bacterial cause of encephalitis (n=4), patients with suspected ABM but no agent detected (n=2), and patients with CSF WBC>5 with other diagnoses (n=17) were excluded in this present study in order to create groups with well-defined conditions (due to the explorative character of the study).

[b]patients with <5 WBC in CSF and agent detected were excluded; *Treponema pallidum* (n=1), Parvovirus B19 positive PCR in CSF (n=1), cerebral abscess with *Streptococcus intermedius* (n=1) and cysticercosis (n=1). None of these patients fulfilled the case definitions in present study(see Additional file1, Table S1).

[c]patients with comorbidity were excluded; 1. Delirium (n=18), 2. psychiatric disorder, acute or chronic (n=14), 3. Parkinson's disease (n=2), Huntington's disease (n=1), tumor cerebri (n=3), 4. dementia (n=7), 5. epileptic seizures or epilepsy in history (n=13), 6. cerebrovascular accident (CVA, n=1), transient global amnesia (TGA, n=3), 7. septicemia (n=6). Several patients had more than one exclusion criteria in the control group.

B. Flow chart of various analyses of the study population
