# Supplementary material for: High neopterin and IP-10 levels in cerebrospinal fluid are associated with neurotoxic tryptophan metabolites in acute central nervous system infections
Source: J Neuroinflammation. 2018 Nov 23;15:327. doi: 10.1186/s12974-018-1366-3 (PMC6260858; doi:10.1186/s12974-018-1366-3)
Supplement: Supplementary file 3 — Table S2. Cytokine levels in CSF and serum. (PDF 215 kb) [file 12974_2018_1366_MOESM3_ESM.pdf]

Table S2 Cytokine and chemokine profile in CSF and serum

Cytokine/ Chemokine	Encephalitis (n=10)	ASM (n=25)	ABM (n=6)	Controls (n=42)	p-value[a]
**CSF**					
TNF	14 (4.2, 56)[b]	95 (25, 159)[b,c]	368 (89, 15383)[b,c]	1.6 (0.5, 2.7)	**<0.001**
IL-1β	0.5 (0.2, 1.4)[b]	3.2 (1.0, 5.1)[b,c]	19 (3.3, 1538)[b,c,d]	0.1 (0.1, 0.2)	**<0.001**
IL-1Ra	17 (9.5, 322)[b]	243 (52, 657)[b,c]	2402 (94, 7038)[b,c]	2.6 (1.7, 3.8)	**<0.001**
IL-2	0.1 (0.0, 1.2)	3.2 (1.0, 13)[b,c]	14 (1.4, 32)[b,c]	0.4 (0.0, 0.7)	**<0.001**
IL-4	0.7(0.3, 1.9)[b]	2.4 (0.5, 5.1)[b,c]	12 (3.5, 19)[b,c,d]	0.1 (0.1, 0.3)	**<0.001**
IL-6	174 (24, 431)[b]	1706 (693, 4810)[b,c]	31787 (2577, 41778) [b,c,d]	6.7 (3.3, 22)	**<0.001**
IL-7	4.9 (3.4, 7.9)[b]	5.0 (3.2, 7.4)[b]	10.4 (5.4, 19)[b,d]	1.6 (0.7, 3.1)	**<0.001**
IL-8	92 (35, 322)[b]	398 (120, 1217)[b]	666 (162, 15432)[b]	15 (9.4, 24)	**<0.001**
IL-9	3.9 (3.0, 6.7)[b]	6.5(3.0, 11)[b]	40 (6.7, 86)[b,c,d]	2.1 (1.4, 2.8)	**<0.001**
IL-10	2.5 (1.7, 12)[b]	22 (3.6, 78)[b,c]	165 (7.9, 435)[b,c]	1.2 (0.7, 1.5)	**<0.001**
IL-12p70	1.0 (0.3, 2.0)	2.5 (0.9, 4.4)[b,c]	9.8 (5.2, 23)[b,c,d]	0.5 (0.3, 1.0)	**<0.001**
IL-13	1.6 (1.1, 2.5)	5.6 (3.8, 8.2)[b,c]	6.6 (4.8, 8.5)[b,c]	1.6 (0.3, 5.1)	**<0.001**
IL-15	8.2 (5.6, 13)[b]	17 (5.1, 26)[b]	38 (14, 75)[b,c,d]	3.7 (2.9, 5.3)	**<0.001**
IL-17A	3.7 (0, 20)	24 (3.2, 51)[b,c]	296 (17, 516)[b,c,d]	5.0 (0.7, 8,6)	**<0.001**
MCP-1	226 (74, 563)	208 (89, 644)[b]	555 (344, 1675)[b]	94 (69, 144)	**0.001**
MIP-1α	1.1 (0.8, 3.9)[b]	3.7 (1.3, 7.8)[b,c]	68 (3.6, 811)[b,c,d]	0.2 (0.1, 0.3)	**<0.001**
MIP-1β	11 (6.4, 17)[b]	27 (8.4, 54)[b]	551 (71, 1391)[b,c,d]	4.3 (2.6, 7.0)	**<0.001**
IP-10	3004 (1847, 27292)[b]	29887 (5803, 33469)[b,c]	26820 (4425, 32658)[b]	559 (158, 1606)	**<0.001**
G-CSF	43 (7.7, 121)[b]	173 (81, 330)[b,c]	2078 (122, 13888)[b,c]	5.9 (1.2, 31)	**<0.001**
GM-CSF	7.1 (0.6, 15)[b]	14 (7.6, 22)[c]	37 (8.4, 58)[b,c]	13 (7.2, 17)	**0.021**
VEGF	21 (11, 33)	41 (14, 77)[b]	146 (101, 301)[b,c,d]	21 (4.5, 30)	**<0.001**
Eotaxin	31 (16, 67)[b]	128 (80, 241)[b,c]	362 (109, 598)[b,c]	8.2 (0.5, 17)	**<0.001**
**Serum**					
IFN-γ	22 (21, 32)	24 (18, 30)	26 (18, 44)	21 (17, 29)	0.608
TNF	72 (65, 92)	83 (67, 97)	103 (84, 193)[b]	68 (51, 88)	**0.041**
IL-1β	2.5 (2.0, 3.2)	2.5 (1.8, 3.4)	3.4 (2.3, 7.4)	2.5 (1.9, 3.1)	0.295
IL-1Ra	81 (60, 272)	73 (62, 114)[b]	274 (178, 4493)[b,c,d]	151 (85, 236)	**0.005**
IL-4	4.5 (3.4, 5.7)	4.1 (3.5, 4.7)	5.1 (3.6, 9.3)	4.2 (3.1, 5.1)	0.511
IL-6	16 (8.9, 27)	10 (4.7, 22)	164 (57, 1596)[b,c,d]	19 (7.4, 30)	**0.002**
IL-8	19 (14, 36)	14 (11, 18)	40 (19, 178)[b,d]	16 (12, 20)	**0.015**
IL-9	59 (47, 91)	47 (39, 57)	63 (44, 79)	49 (40, 58)	0.150
IL-10	16 (7.6, 41)	9.4 (6.4, 14)	26 (8.7, 54)	10 (6.6, 21)	0.153
IL-12p70	37 (14, 72)	25 (18, 45)	37 (16, 54)	25 (15, 41)	0.806
IL-13	12 (2.5, 18)	6.1 84.5, 12)	3.7 (2.6, 4.8)	4.4 (3.5, 8.2)	0.142
IL-17A	188 (129, 382)	131 (88, 176)	190 (113, 251)	131 (92, 208)	0.240
MCP-1	62 (16, 87)	20 (11, 36)	46 (32, 362)	30 (16, 59)	0.103
MIP-1α	5.1 (3.8, 9.3)	4.5 (3.9, 6.3)	5.1 (4.7, 7.0)[b]	4.1 (3.3, 4.9)	**0.044**
MIP-1β	128 (94, 200)	81 (54, 124)	122 (81, 289)	103 (81, 166)	0.064
IP-10	377 (175, 1139)	187 (114, 354)	969 (537, 7485)[d]	318 (127, 1375)	**0.035**
VEGF	189 (23, 379)	132 (89, 250)	247 (72, 411)	148 (86, 233)	0.767
Eotaxin	25 (17, 30)	23 (18, 37)	25 (18, 38)	29 (19, 42)	0.488
PDGF	334 (242, 572)	338 (240, 526)	300 (25, 566)	361 (248, 562)	0.833
Rantes	13972 (11673, 19654)	16984 (12039, 19522)	14148 (10534, 15944)	14089 (10594, 19544)	0.642

Data shown are median (IQR), all units are in pg/ml

[a]p values for one way analysis of variance (Kruskal Wallis)

[b]p<0.05 for analysis with Mann-Whitney U test (MWU) in comparison with control group

[c]p<0.05 for analysis with MWU in comparison with encephalitis

[d]p<0.05 for analysis with MWU in comparison with ASM.
