# Supplementary material for: High neopterin and IP-10 levels in cerebrospinal fluid are associated with neurotoxic tryptophan metabolites in acute central nervous system infections
Source: J Neuroinflammation. 2018 Nov 23;15:327. doi: 10.1186/s12974-018-1366-3 (PMC6260858; doi:10.1186/s12974-018-1366-3)
Supplement: Supplementary file 4 — Table S3. Correlations of cytokines in CSF with serum levels, CSF WBC, albumin ratio and KYN/TRP ratio. (PDF 197 kb) [file 12974_2018_1366_MOESM4_ESM.pdf]

Table S3 Correlations of cytokines in CSF with serum level, CSF WBC, albumin ratio and KYN/TRP ratio

Cytokine/chemokine	Serum level (n=36)	CSF WBC (n=41)	Albumin ratio (n=34)	KYN/TRP ratio in CSF (n=28)
TNF	**0.37(0.03)**	**0.65(<0.001)**	**0.48(0.004)**	**0.54(0.003)**
IL-1b	0.24(0.17)	**0.65(<0.001)**	**0.50(0.003)**	**0.54(0.003)**
IL-1Ra	**0.35(0.03)**	**0.63(<0.001)**	**0.52(0.002)**	**0.64(<0.001)**
IL-4	0.18(0.30)	**0.57(<0.001)**	**0.44(0.009)**	**0.53(0.004)**
IL-6	**0.47(0.004)**	**0.67(<0.001)**	**0.50(0.003)**	**0.48(0.011)**
IL-8	0.17(0.33)	**0.48(0.001)**	**0.50(0.002)**	**0.47(0.013)**
IL-9	0.13(0.45)	**0.36(0.02)**	**0.45(0.007)**	**0.58(0.001)**
IL-10	0.09(0.62)	**0.62(<0.001)**	**0.49(0.003)**	**0.70(<0.001)**
IL-12p70	-0.04(0.82)	**0.62(<0.001)**	**0.56(0.001)**	**0.50(0.007)**
IL-13	0.12(0.51)	**0.34(0.03)**	**0.42(0.01)**	**0.42(0.026)**
IL-17A	0.03(0.87)	**0.46(0.003)**	**0.35(0.04)**	**0.55(0.002)**
MCP-1	**0.35(0.04)**	0.11(0.50)	0.30(0.08)	0.07(0.73)
MIP-1α	0.22(0.20)	**0.59(<0.001)**	**0.53(0.001)**	**0.55(0.003)**
MIP-1β	0.11(0.51)	**0.35(0.03)**	**0.37(0.03)**	**0.46(0.014)**
IP-10	0.05(0.76)	**0.33(0.04)**	0.25(0.16)	**0.70(<0.001)**
VEGF	0.03(0.88)	**0.53(<0.001)**	**0.50(0.003)**	**0.42(0.026)**
Eotaxin	0.32(0.06)	**0.64(<0.001)**	**0.48(0.004)**	**0.53(0.004)**

Data are Spearman rank correlation coefficients (Rho (p-value) for patients with CNS infection of measurable cytokines and chemokines in CSF with serum level of same metabolite, CSF white blood cell count (CSF WBC), albumin ratio (CSF albumin/serum albumin) and KYN/TRP ratio (as an expression of IDO activity in the kynurenine pathway).
