# Supplementary material for: High neopterin and IP-10 levels in cerebrospinal fluid are associated with neurotoxic tryptophan metabolites in acute central nervous system infections
Source: J Neuroinflammation. 2018 Nov 23;15:327. doi: 10.1186/s12974-018-1366-3 (PMC6260858; doi:10.1186/s12974-018-1366-3)
Supplement: Supplementary file 5 — Table S4. KP metabolites in CSF and serum. (PDF 198 kb) [file 12974_2018_1366_MOESM5_ESM.pdf]

Table S4 CSF and serum levels of KP metabolites

KP metabolite	Encephalitis (n=10)	VM (n=12)	ABM (n=6)	Controls (n=22)	p-value[a]
**CSF**					
TRP[e]	1.95 (0.7, 2.5)	0.4 (0.4, 0.9)[b,c]	3.3 (0.4, 4.2)[d]	1.95 (1.6, 2.3)	**<0.001**
KYN[e]	0.1 (0.1, 0.5)[b]	0.4 (0.4, 1.0)[b,c]	0.8 (0.3, 1.8)[b,c]	0.04 (0.02, 0.05)	**<0.001**
KYNA	6.9 (2.7, 11)[b]	7.9 (3.3, 23)[b]	37 (10, 93)[b,c,d]	2.1 (1.7, 3.1)	**<0.001**
AA	13 (7.5, 28)[b]	17 (11, 58)[b]	47 (10, 78)[b]	3.9 (2.9, 5.6)	**<0.001**
3-HK	22 (5.4, 30)[b]	20 (10, 29)[b]	23 (16, 78)[b]	4.3 (3.1, 4.4)	**<0.001**
3-HAA	8.6 (2.4, 27)[b]	25 (10, 121)[b,c]	45 (27, 103)[b,c]	< LOD	**<0.001**
QA	445 (236, 744)[b]	279 (128, 988)[b]	619 (166, 3180)[b]	21 (14, 42)	**<0.001**
PIC	31 (23, 91)[b]	25 (16, 31)	47 (30, 118)[b,d]	20 (13, 29)	**0.008**
Neopterin	97 (44, 149)[b]	100 (76, 123)[b]	85 (45, 146) [b]	19 (16, 25)	**<0.001**
KYN/TRP ratio (IDO)[f]	61 (42,725)[b]	993 (492,249)[b,c]	295 (66,3953)[b]	16 (12,20)	**<0.001**
**Serum**					
TRP[e]	47 (31, 57)	56 (45, 63)	22 (11, 33) [b,d]	55 (47, 64)	**0.001**
KYN[e]	1.4 (1.1, 3.3)	1.3 (1.1, 1.7)	2.1 (1.2, 5.1)	1.6 (1.2, 1.9)	0.302
KYNA	49 (38, 69)	35 (26, 53)	45 (39, 212)	36 (24, 46)	0.074
AA	20 (12, 31)	14 (12, 16)	17 (14, 92)	15 (12, 24)	0.290
3-HK	51 (29, 110)	43 (32, 53)	93 (50, 359)	50 (39, 61)	0.228
3-HAA	38 (21, 84)	25 (20, 39)	48 (27, 80)	27 (23, 42)	0.407
QA	413 (234, 1367)	294 (243, 392)	1185 (480, 3222)	377 (298, 583)	0.065
PIC	36 (28, 171)	38 (28, 55)	76 (60, 133)	32 (22, 52)	0.053
Neopterin	31 (18, 61)	24 (21, 30)	67 (41, 122)	30 (20, 43)	0.128
KYN/TRP ratio (IDO)[f]	28 (21,94)	21 (19,31)[b]	114 (50,448)[b,d]	29 (25,34)	**0.009**

Data shown are median (IQR)

[a]p values for one way analysis of variance (Kruskal Wallis)

[b]p<0.05 for analysis with Mann-Whitney U test (MWU) in comparison with control group

[c]p<0.05 for analysis with MWU in comparison with encephalitis

[d]p<0.05 for analysis with MWU in comparison with VM

[e]Units are micromoles/L (μM), for all other metabolites units are in nanomoles/L (nM)

[f]KYN/TRP ratio is calculated as KYN(nmol)/TRP(μmol).
