# Supplementary material for: High neopterin and IP-10 levels in cerebrospinal fluid are associated with neurotoxic tryptophan metabolites in acute central nervous system infections
Source: J Neuroinflammation. 2018 Nov 23;15:327. doi: 10.1186/s12974-018-1366-3 (PMC6260858; doi:10.1186/s12974-018-1366-3)
Supplement: Supplementary file 6 — Figure S2. Serum levels of KP metabolites. (PDF 81 kb) [file 12974_2018_1366_MOESM6_ESM.pdf]

Fig. S2

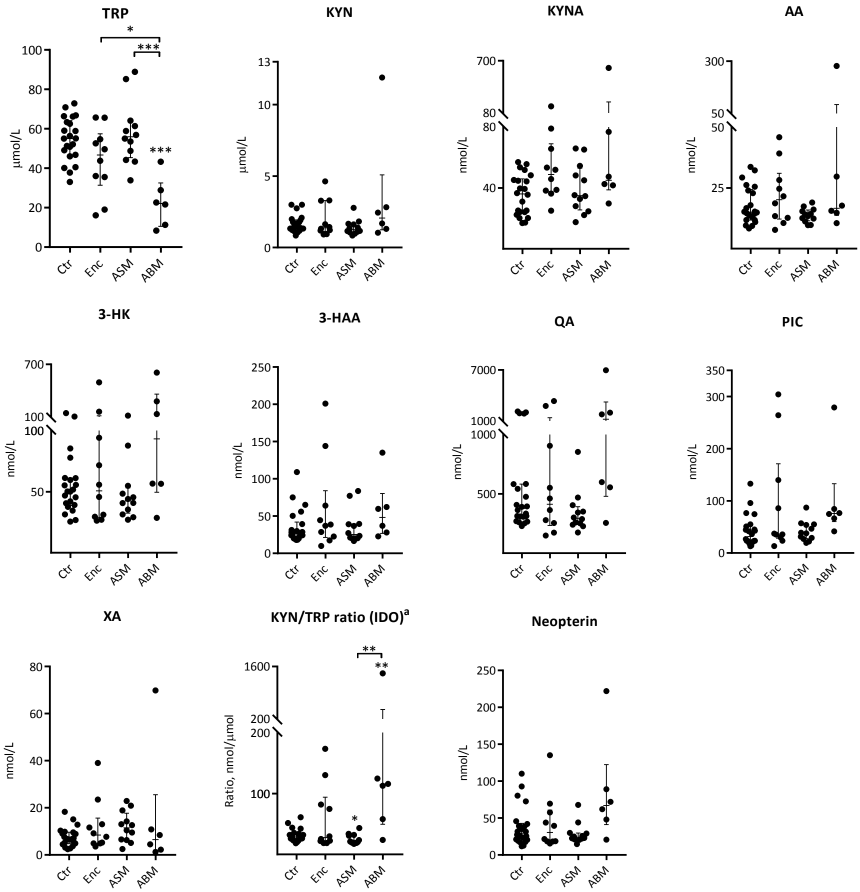

Kynurenine metabolites and enzymes in serum of patients with encephalitis (Enc, n=10), viral meningitis (VM, n=12) and bacterial meningitis (ABM, n=6) in comparison with controls (Ctr, n=22). Data shown are medians with IQR. If significant in analysis of v ariance with the Kruskal Wallis test, comparisons of two groups were analysed by Mann -Whitney U test.

Asterisks above patient groups indicate significant difference vs controls, asterisks above horizontal lines indicate significant differences between individual groups (Mann-Whitney U tests):*p<0.05, **p<0.01 and ***p<0.001.

[a] KYN/TRP ratio is calculated as KYN (nmol)/TRP(µmol)
