# Supplementary material for: High neopterin and IP-10 levels in cerebrospinal fluid are associated with neurotoxic tryptophan metabolites in acute central nervous system infections
Source: J Neuroinflammation. 2018 Nov 23;15:327. doi: 10.1186/s12974-018-1366-3 (PMC6260858; doi:10.1186/s12974-018-1366-3)
Supplement: Supplementary file 7 — Table S5. CSF/serum ratios of KP metabolites and the correlation with CSF WBC and albumin ratio. (PDF 194 kb) [file 12974_2018_1366_MOESM7_ESM.pdf]

Table S5 CSF/serum ratios of KP metabolites (A) and the correlation with CSF WBC and albumin ratio (B)

A.

CSF/serum ratio	Encephalitis (n=10)	VM (n=12)	ABM (n=6)	Controls (n=22)	P – value[a]
TRP[e]	0.043 (0.027,0.063)	0.0080 (0.0063,0.017) [b,c]	0.13 (0.038,0.24) [b,d]	0.037 (0.028,0.053)	**<0.001**
KYN	0.098(0.066,0.26)[b]	0.40(0.32,0.61)[b,c]	0.36(0.13,0.65)[b]	0.022(0.017,0.028)	**<0.001**
KYNA	0.12(0.077,0.19)[b]	0.21(0.090,0.56)[b]	0.27(0.14,2.19)[b]	0.076(0.051,0.095)	**<0.001**
AA	0.66(0.48,0.92)[b]	1.24(0.85,3.60)[b,c]	1.27(0.38,4.02)[b]	0.24(0.20,0.32)	**<0.001**
3-HK	0.25(0.15,0.37)[b]	0.38(0.25,0.76)[b]	0.28(0.18,0.43)[b]	0.078(0.069,0.10)	**<0.001**
3-HAA	0.20(0.053,0.49)[b]	0.90(0.37,3.22)[b,c]	0.88(0.41,2.71)[b,c]	na	**<0.001**
QA	0.88(0.21,1.50)[b]	0.75(0.39,3.37)[b]	0.64(0.20,1.64)[b]	0.050(0.036,0.064)	**<0.001**
PIC	0.72(0.60,0.89)	0.69(0.45,0.72)	0.72(0.55,0.82)	0.56(0.46,0.79)	0.51
Neopterin	2.2(1.46,4.03)[b]	4.43(2.33,5.66)[b]	0.99 (0.86,2.33)[b,d]	0.62(0.48,0.75)	**<0.001**

B.

CSF/serum ratio	CSF WBC (n=28)	CSF albumin/serum albumin (n=23)
TRP[e]	-0.24(0.22)	0.25(0.24)
KYN	**0.44(0.02)**	0.25(0.25)
KYNA	0.21(0.28)	0.36(0.10))
AA	**0.40(0.04)**	0.14(0.51)
3-HK	**0.45(0.02)**	0.32(0.14)
3-HAA	**0.64(<0.001)**	0.41(0.052)
QA	**0.39(0.04)**	0.23(0.29)
PIC	0.20(0.31)	**0.56(0.005)**
Neopterin	0.20(0.31)	-0.03(0.91)

A. The relative concentrations of metabolites (tryptophan, kynurenines and neopterin) in CSF versus serum for patients with CNS infection given as CSF/serum ratio, indicating a lower level of KP metabolites in CSF compared with serum for most metabolites. Data shown are median (IQR).

B. Correlation of metabolite CSF/serum ratio with CSF white blood cell count (CSF WBC) and albumin ratio (CSF albumin/serum albumin) for patients with CNS infection given as Spearman Rho (p- values).

[a]p values for one way analysis of variance (Kruskal Wallis)

[b]p<0.05 for analysis with Mann-Whitney U test (MWU) in comparison with control group

[c]p<0.05 for analysis with MWU in comparison with encephalitis

[d]p<0.05 for analysis with MWU in comparison with VM.

[e]TRP levels below lower level of detection (LOD) for 9 patients with CNS infection were adjusted to this value (0.4 µM) for calculation of the TRP ratio
